# Learning digital skills online: empowering older adults through one-to-one, online digital training provision

**DOI:** 10.3389/fpsyg.2023.1122277

**Published:** 2023-05-05

**Authors:** Gemma Wilson-Menzfeld, Jessica Raven Gates, Mary Moreland, Helen Raw, Amy Johnson

**Affiliations:** ^1^Department of Nursing, Midwifery, and Health, Faculty of Health and Life Sciences, Northumbria University, Newcastle upon Tyne, United Kingdom; ^2^War Widows’ Association of Great Britain, London, United Kingdom

**Keywords:** digital, critical geragogy, older adult, skill building, digital skill development, facilitators, barriers, training

## Abstract

**Introduction:**

Digital exclusion, through lack of access and poor digital skills, can have an adverse impact on daily living. Not only did the COVID-19 pandemic dramatically impact the necessity of technology in our daily lives, but also reduced the availability of digital skills programmes. This study aimed to explore perceived facilitators and barriers of a digital skills programme that was delivered remotely (online) and to reflect on this form of training as a possible alternative to traditional face-to-face models.

**Methods:**

Individual interviews were carried out with programme participants and the programme instructor.

**Results:**

Two themes were generated from this data: (a) Creating a unique learning environment; and (b) Encouraging further learning.

**Discussion:**

Barriers to digital delivery were evident, however, the individual and personalized delivery empowered participants within their own learning, supporting individuals to learn skills relevant to them and to continue their digital learning journey.

## Introduction

The COVID-19 pandemic heightened the ubiquity of online participation across our society. Individuals changed their ways of working, teaching and learning n, communicating with one another, and ways of accessing services such as banking (Martin, 2020), booking GP appointments (Clarke et al., 2020), and participating in online exercise classes (Wilson-Menzfeld et al., 2022), to name but a few. Not everyone was able to ride the digital wave and navigate this rapid shift, and this “digital divide” resulted in a radical increase in inequalities across the UK and internationally (Bower et al., 2021). Inequity of digital use comprises multiple levels; access (including access to the internet and other material access, for example, digital devices) (Van Deursen and Van Dijk, 2019); digital skills; and not recognizing the benefits of using the internet (Van Deursen and Helsper, 2015).

Although pandemic restrictions have eased across the globe, many services remain online, maintaining inequities for those who remain offline and digitally excluded. While acknowledging the lack of evidence in this area, Honeyman et al. (2020) theorized that digital exclusion could influence health inequalities directly; i.e., the inability to access digital-based health improving services or resources, and indirectly; limited access to wider determinants of health, such as housing and benefits prospects which are offered through digital means. Consequently, this impacts the individual’s behavior and leads to unmet need, which in turn can negatively impact health and wellbeing. It is critical that digital transformations, including, digital health transformations, must be designed with health equity at the forefront (Kickbusch et al., 2021; Van Kessel et al., 2022a).

Inequalities throughout the life course increase the risk of digital exclusion in later life (Wilson-Menzfeld and Brittain, 2022). However, while there has been a rise in internet use from those over 75 in the last decade (Eurostat, 2017; Office for National Statistics [ONS], 2018), older adults still use the internet to a lesser extent than younger generations and are more likely to be considered ‘digitally excluded’ (Age UK, 2018). For instance, in a recent exploratory analysis of Eurostat data, Van Kessel et al. (2022b) reported that only 7.87% of adults aged between 65 and 74 years old reported above basic digital skills compared to 60.35% of those aged 16–24 years old. The presence of digital skills for older adults is critical to improving digital inclusion. Aging has generally been considered as a social construct, with perceptions being influenced by culture, societal expectations, and individual experiences (Chonody and Teater, 2016). NHS England (n.d.) generally consider someone over the age of 65 to be an older person. Traditionally, in the UK, 65 years of age was the official retirement age for men and the age they could utilize their State Pension, therefore this long has been used as a threshold for older age (Office for National Statistics [ONS], 2019). However, due to changes to working patterns, changes to the official retirement age and people living longer lives, the threshold of considering someone 65 years of age an older adult may soon begin to shift (Office for National Statistics [ONS], 2019). For the purposes of this paper, the definition of older adults being 65 years and older, used by NHS England, is followed.

**FIGURE 1:**
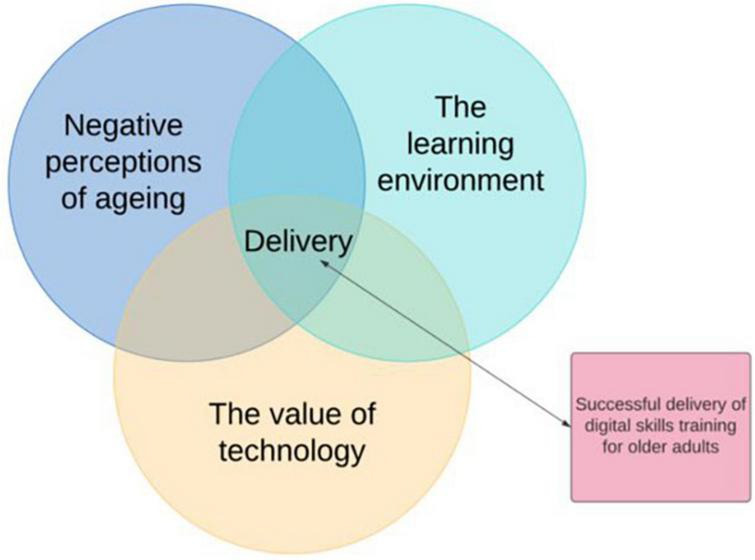
Theoretical model highlighting components for a successful delivery of digital skills to older adults (Wilson-Menzfeld et al., 2021; Gates and Wilson-Menzfeld, 2022).

The development of skills in later life, including digital skills, can be both fulfilling and empowering (Withnall, 2009). Geragogy, and Critical Geragogy, is a distinct part of Pedagogy, a learning theory focused on learning in later life (Formosa, 2002, 2011, 2012; Findsen and Formosa, 2012). Rather than focusing on the psychological deficit model, Geragogy and Critical Geragogy recognize older adults’ distinct physical, emotional, and social learning needs, and aims to empower learners through self-directed and self-led learning (Lebel, 1978; Formosa, 2002; Wright and Wright, 2016). A recent systematic narrative review examined the role of Geragogy and Critical Geragogy in the delivery of digital skills programmes for middle and older age adults (Gates and Wilson-Menzfeld, 2022). Whilst only one of the 17 papers explicitly referred to learning theory, the review highlighted the importance of three intersecting components that impact digital skills training for older adult; negative perceptions of aging, the learning environment, and the value of technology (Braun and Clarke, 2006; Gates and Wilson-Menzfeld, 2022; Figure 1).

Self-efficacy can impact the learning process and the development of new skills. The learner’s perception of their own abilities and capabilities can influence motivation to complete learning-based tasks, effort placed in learning, as well as the likelihood to continue in the event of obstacles (Bandura, 1977, 1982). Self-fulfilling prophecy, in contrast, incorporates and acknowledges the influence of the teacher’s expectations of the learner’s abilities on their development and academic behaviors (Jussim, 1986). Digital skills can be impaired by both self-efficacy and self-fulfilling prophecy. Self-efficacy judgments are impacted by prior internet experience, internet use, and outcome expectancies (Eastin and LaRose, 2000) and an individual’s internet efficacy impacts willingness to use digital services (Tetri and Juujärvi, 2022). Furthermore, existing aging stereotypes toward older adults’ digital and internet use can exacerbate an individual’s own feelings toward technology use (Comunello et al., 2022).

Gates and Wilson-Menzfeld (2022)’s systematic review highlighted that older adults often hold negative perceptions of their own aging and demonstrated how this impacted learning digital skills. This aging stereotype must be challenged when initiating digital learning programmes through the promotion of individual learning styles and reflexive learning. Facilitating empowerment through the learning environment was important to support digital skills training. This involved the recognition of distinct needs, building rapport with learners in a safe space, and ensuring delivery aligned with learner expectations and needs. Finally, an individual’s own needs must be central to learning through personalization; this required continual check-ins and reflection. Taken together, these factors can improve the implementation and outcomes of digital skills programmes, improving sustainability of programmes over time. It is fundamental that learning theories, such as Critical Geragogy, are embedded in digital skills programmes to remove the misconception that ‘*any type of learning will do’* (Gates and Wilson-Menzfeld, 2022).

The War Widows’ Association (WWA) is a registered charity with 1,941 members (as of November 2021). To be a full member, an individual must receive/have received a War Widows’ Pension or Armed Forces Compensation Scheme 2005 payments. Any individual interested in the welfare of War Widow(er)s or in supporting the aims of the WWA can become an associate member. The WWA recognized issues of loneliness and social isolation across their membership, along with the desire of members to be connected to other members throughout the UK. Working with Northumbria University, the WWA designed a digital intervention, the War Widows InTouch (WW.it) programme, to address these needs and to connect older war widow(er)s (over 65 years old) at both a national and local level (Wilson-Menzfeld et al., 2021). The WW.it programme also aimed to increase digital access, digital confidence, and digital skills, as well as reducing fear and the impact of aging stereotypes on digital learning. Utilizing Critical Geragogy as an underpinning learning theory, WW.it aimed to provide a personalized intervention which encouraged older war widows to take an active role in digital skills training, working collaboratively with the instructor throughout (Wilson-Menzfeld et al., 2021). To accomplish this, members of the WWA were given iPads and/or iPad training (Wilson-Menzfeld et al., 2021). This project took lessons from “Project Semaphore” which was carried out by the Royal Naval Association and had similar project aims (Royal Naval Association, n.d.). However, due to the COVID-19 pandemic, the implementation and running of the WW.it programme changed significantly. Initially the programme was intended to be completed face-to-face, and in a group setting, but was ran remotely, online, and in a one-to-one setting. This training model allowed individuals to receive digital skills training at a time when the use of technology was being perceived as a fundamental part of everyday life. However, this is a very different model of training than had been previously considered.

Due to the unique mode of digital skills delivery of the WW.it programme, this study aimed to explore the perceived facilitators and barriers of the WW.it online digital skills programme from the perspective of both the instructor and participants. In doing so, this study aims to reflect on this form of training (i.e., remote, online, one-to-one training) as a possible alternative to traditional face-to-face models.

## Materials and methods

### Design

This study is part of a larger, two-phase project which involved a mixed-method explanatory sequential design (Creswell et al., 2011). Mixed methods designs are typically chosen for evaluation studies to assess the impact of a programme, whilst also providing an in-depth view of the participant experiences to provide a more complete picture (Creswell and Clark, 2017). This mixed methods design, underpinned by Pragmatism (Feilzer, 2010; Morgan, 2014), allowed the research team to identify the self-reported impact of the WW.it programme, whilst gathering in-depth information regarding the implementation (see Wilson-Menzfeld et al., 2021 for full evaluation). This paper will focus on the data collected as part of semi-structured interviews across both Phase One and Phase Two only. Quantitative analysis from this mixed methods study is presented elsewhere (Wilson-Menzfeld et al., 2021). Ethical approval was received from Northumbria University’s ethical approval system (ref: 120.3305). This study adhered to the UK Government’s COVID-19 rules and Northumbria University’s guidance on social distancing and completing face-to-face research.

### Participants

The WWA supported recruitment of their members into the WW.it programme through advertisement in an Association newsletter which is regularly mailed to all members. Those interested in taking part in the WW.it programme responded directly to the advertisement. Recipients of the WW.it programme were then invited to take part in the evaluation study. Participation in the research evaluation was voluntary and did not impact selection onto the WW.it programme (i.e., receipt of iPads and/or training).

To participate in the evaluation, participants needed to be members, or associate members, of the WWA and be aged 65 years or above. There were no other eligibility requirements for the study. All of the participants were female due to the membership demographics at the WWA being predominately female. No specific criteria for digital skills was taken. Participants were recruited from across the UK. A purposive recruitment approach was taken to increase inclusion of demographics such as age, location, previous military service, and length of time as a member of the WWA.

Seventeen participants chose to participate in semi-structured interviews in Phase one. Twelve of the same cohort also completed semi-structured interviews at Phase two (Table 1).

**TABLE 1:** Participant characteristics in phase one and phase two interviews*.

	Phase one (*n* = 17)	Phase two (*n* = 12)
Age	66–90 years (Mean = 78.24, SD = 7.28)	66–90 years old (Mean = 77.75, SD = 7.65)
Location	Greater London (18%)	Greater London (17%)
	Northern England (24%)	Northern England (33%)
	Mid England (12%)	Mid England (8%)
	Southern England (35%)	Southern England (33%)
	Scotland (6%)	Scotland (8%)
	Northern Ireland (6%)	Northern Ireland (33%)
Marital status	Married, civil partnership or co-habiting (18%)	Married, civil partnership or co-habiting (25%)
	Widowed (77%)	Widowed (75%)
Children	Yes (82%)	Yes (83%)
	No (18%)	No (17%)
Living status	Lived alone (76%)	Lived alone (67%)
	Lived with others (24%)	Lived with others (33%)
Occupation	Retired (33%)	Retired (33%)
	Employed part-time (13%)	Employed part-time (25%)
	Unpaid/voluntary work (31%)	Unpaid/voluntary work (33%)
	Unemployed/not currently looking for work (15%)	Unemployed/not currently looking for work (8%)

*Those options which equated to 0% are not shown.

Phase two also involved an interview with the instructor who delivered this project and the iPad training (*N* = 1).

### The WW.it programme

The WW.it programme was a personalized, remote, one-to-one digital skill building programme, featuring access to an individual instructor over the phone and via Zoom. Participants in the WW.it programme had access to support from the instructor throughout project duration (1 year) and 6 months following its conclusion.

From the onset, it was clear that participants held limited experience or knowledge of digital applications, therefore the WW.it programme was adjusted according to their individual learning needs, prior digital knowledge, and motivations for joining the programme, which was assessed in the early sessions. As a result, content was personalized to each individual. Despite this, topics relating to turning on the device, using apps, taking photos and online security and safety were covered with all participants.

Initially, the WW.it programme was going to be completed in-person and via UK-based Apple stores. However, following the COVID-19 pandemic and UK nationwide lockdowns resulting in temporary business closures, this was moved to a fully remote one-to-one training session with a sole instructor. Group training was not possible due to difficulty supporting multiple participants to join the video call, particularly due to their limited baseline digital skills. The role of the instructor was not initially intended to provide the training and therefore did not undergo specific IT training themselves. Despite this, their IT experience and competency as a lifelong Apple user was assessed as sufficient during the interview for the role, particularly regarding the participant’s baseline digital knowledge. Additionally, coming from the military bereaved population themselves provided them with a shared understanding with the participants and the ability to quickly build rapport.

### Materials

A semi-structured interview schedule was developed using the findings from a systematic narrative review conducted by some members of the research team (Gates and Wilson-Menzfeld, 2022) and from findings of the survey (as part of the wider mixed methods study; Wilson-Menzfeld et al., 2021). Contents of the survey included: demographic information; information about their membership to the WWA and other organizations; use of and attitudes toward technology; current social connections; and the impact of COVID-19 on their social connections and technology use. The survey responses also allowed personalization of the interview schedule for each participant.

While the systematic review findings and the survey results guided these conversations, the interviews were semi-structured in nature and remained flexible in reaching the goal of understanding how participants use technology and how they would benefit from the WW.it programme. The Phase One interview guide incorporated the concepts of value, underlying aims of participating in the WW.it programme, as well as feelings toward technology. The Phase Two interview guide also prompted discussion of negative perceptions of aging and the learning environment having completed the WW.it programme.

### Procedure

Individuals who had volunteered to join the WW.it programme were invited to participate in the evaluation. Those who wished to participate were contacted the research team and a consent form was posted to them. They were able to opt into taking part in a semi-structured virtual or telephone interview by providing contact details to the research team. A member of the research team (JRG/AJ/GWM/MM) contacted participants to complete the interview. Interviews were completed prior to (Phase one) and following receipt of (Phase two) the iPad/iPad training. Interviews ranged from 15 to 60 min^1^, were audio recorded and transcribed anonymously.

### Data analysis

Data was analysed using Braun and Clarke’s reflexive, inductive Thematic Analysis (Braun and Clarke, 2006, 2013, 2019), facilitated by The NVIVO 12 software package. Whilst not atheoretical, a key strength of Thematic Analysis is that it’s not aligned with a specific methodology or philosophical underpinning, demonstrating suitability for the pragmatic approach used within this wider project (Braun and Clarke, 2006).

Noting that authors tend to assume Thematic Analysis as a singular method, Braun and Clarke (2021) present their work as a “family of methods,” not as a “recipe” (Braun et al., 2022), comprised of similarities but key differences relating to coding methods, developing themes and conceptualizing results. One of these approaches is reflexive Thematic Analysis which is suitable for experiential epistemologies, and inductive analytic processes, as is carried out in this study (Braun and Clarke, 2021). Utilizing reflexive Thematic Analysis, this study analysed data using interpretative, reflexive processes (Braun and Clarke, 2021). Whilst using Critical Geragogy as a lens to develop the programme itself, data was analysed inductively, from the “bottom up” without a coding framework, before being abstracted and considered through its relationship to this learning theory.

A key component of reflexivity is acknowledging the role of the researcher, and the influence their positionality and philosophical underpinnings on the data analysis. Ravitch and Riggan (2012) outline the influence of the researcher’s personal experiences, previous literature, and theoretical and ontological frameworks on developing research questions. Within this study, two members of the research team have lived experiences of military bereavement and therefore brought their own personal perspective to this study. This prior experience guided the research development and facilitated building rapport with the participants.

Braun and Clarke (2006) suggest six stages to facilitate Thematic Analysis: data familiarization, generating initial codes, creating themes, reviewing themes, defining and naming themes, and producing the final product. These stages were used as a tool to help guide the process of Thematic Analysis. In this case, members of the research team read and re-read the transcripts and after familiarization, used the NVIVO software to highlight potentially relevant or interesting quotes. In addition to highlighting quotes, the research team (GWM/JRG) left annotations throughout, as to the reasoning why they believed the quote to be relevant or interesting. These initial annotations then formed the basis of inductively generating the initial codes. These initial codes were then grouped together into categories to create initial themes. At first these categories were highly descriptive, however, upon review, the themes became more conceptual. The research team then discussed these different concepts, and finally decided upon definitions and names for the developing themes. Throughout this process, there was considerable movement back and forth between phases to generate the final themes.

## Results

Two themes were generated from the data: Creating a unique learning environment; and encouraging further learning (Table 2). Each theme is made up of multiple sub-themes.

**TABLE 2:** Themes and sub-themes generated from the data.

Creating a unique learning environment	Personalization
	Building rapport
	The digital vs. in-person environment
Encouraging further learning	The learning journey
	Wider barriers to online participation

It is important to consider the group’s previous experiences with digital technology to contextualize these findings. The vast majority of the cohort had very little, or no, prior experience using digital technology, including the iPad.

*“I will start by saying my answer is a little bit restricted because in all honesty I don’t know what you can do with an iPad [*…*] Once, if you like, I am a bit more aware of what you can do with it, I am assuming that more possibilities will suddenly become available” (P016, Age 79)*.

*“Being shown what it can do, probably that I don’t even know what it can do*…*I haven’t even thought about some things probably” (P023, Age 71)*.

This impacted their expectations of using digital technology and the level of digital training provided.

### Creating a unique learning environment

#### Personalization

Whilst not all online learning environments lend themselves to personalization, the personalized approach adopted by the WW.it programme was perceived as being central to its success. The WW.it training sessions were initially intended to be run as group sessions, however, due to the COVID-19 pandemic, all sessions were completed as remote, one-on-one sessions (unless another family member was present).

This personalization began at initial sign up to the programme. The instructor spent time speaking to each individual and developing a relationship. In doing so, the instructor was able to understand individual motivations for participating and previous experiences of technology use.

*“In a way it is probably good that I have done a lot of the training because I’ve had this kind of sort of phone relationship with a lot of them and had the conversations [*…*] [*…*] I’m slightly different with each one because you know they’re all different and how they are with me [*…*] I kind of bounce back that either same level of energy or you know, if there’s somebody who is really quite into laughing and joking around I will match that” (Instructor)*.

*“It was personalized to me, very much so” (P020, Age 79)*Importantly, for online digital skills delivery, the instructor was able to understand an individual’s barriers to participating in the programme. For example, not having access to broadband, or a second device for online programme delivery. This had implications as to what device the individual received and how the training was completed.

*“She had her sort of standard format that she wanted to cover, but then she adjusted that [*…*] if we got to something, she’d say, do you understand this? [*…*] Are you familiar with it? And sometimes I was familiar with certain aspects. So, we were able not to spend time on those too much” (P032, Age 71)*.

Whilst this resulted in a positive experience for participants, it was labor intensive, and potentially unsustainable for future programmes.

*“ [*…*] I’d allowed sort of 15 min per phone call and you know there were some I was on for an hour and a half” (Instructor)*.

As this programme was delivered on a one-to-one basis, the instructor was able to tailor sessions to individual needs after providing some generic training information. Importantly, this allowed personalization in both learning style and content. A mix of both basic digital skills training and more personalized training materials was seen as beneficial. Not only did this allow individuals to develop the skills they found most useful, but it also added value to the training and participants thought this would be more useful than group training.

*“There was one individual [*…*] she’s got an amazing garden and she loves taking pictures of the flowers, but she didn’t know how to send them to people. So instead of going round two of the simple apps. I went round one of them and then we went straight into camera, and we did over an hour just inside the camera and she was taking various pictures in her house” (Instructor)*.

*“I think if you were trying to do it in a group, it would be difficult, because okay, some things would be common, but for example, my problem with my router password [*…*] it wouldn’t really have been of interest to other people, and it took up so much time” (P016, Age 79*).

As well as recognizing the importance of specific learning content, this one-to-one programme enabled the instructor to recognize the importance of individual differences in learning style and designed the training around this, arguably improving their learning experience and empowering the learners in this process.

*“It’s been nice to see how different people learn” (Instructor)*.

In delivering more tailored content, in a way that most benefited the learner, this tailored approach also allowed accessibility options to be explored for each learner, once more, empowering individuals to utilize their digital device in the best way for them. Some participants found it difficult to interact with the iPad due to sight impairment, hearing impairment, or dexterity issues. Accessibility options were described as one of the greatest benefits of training.

*“And then this is how you can put a screen saver shot on and this is [*…*] how you can make the words, the text larger if you want to” (P032, Age 71)*.

Participants described how the training ‘debunked’ the iPad for them. Many participants described how different the iPad was to the technology they had previously used, e.g., a laptop or desktop computer. This was primarily through lack of on-screen text, which is replaced by apps, jargon, and a touchscreen.

*“it’s about finding a way to make it not sound complicated. Not make them feel stupid, like oh I should have known that but also make it that they actually want to keep learning, because tech stuff [*…*] it is the most boring thing in the world and if you don’t know how to do something” (P001, Age 76)*.

This debunking was facilitated through personalized, one-to-one learning, in which the instructor built up a relationship with each participant and began to understand their own needs and difficulties.

“She focused on all the little symbols. […] the little symbols on the iPad that I didn’t understand and then she taught me about the eBay” (P015, Age 88).

#### Building rapport

Several participants exhibited low confidence with their own digital skills and often made self-deprecating comments about themselves and their abilities.

*“I would like to get my training [*…*] because I really don’t want to be labeled a slow person when I’m not really a slow person” (P019, Age 84)*.

P018 made several negative comments about herself being “*stupid*” and demonstrated extremely low confidence.

*“I’m not that clever” (P018, Age 80)*.

*“I must seem very stupid to you. I’m sorry” (P018, Age 80)*.

It was important for there to be a sense of familiarity between the instructor and learner to recognize these self-held beliefs, and often aging stereotypes, as potential setbacks to learning. In the WW.it programme, there was one instructor throughout the duration of the programme, and this familiarity helped to develop the relationship between instructor and learner, facilitating learning.

*“I liked her on the phone. I think it helps to like the person” (P001, Age 76)*.

This familiarity was especially important to those who were anxious about using technology and starting the training programme.

*“She understood and she knew everything, and she really was a benefit and of course the mistakes that I was making there, in my training, but that helped because the same thing was happening when I was trying to work it on my own and for her to explain what all these other things were” (P015, Age 88)*.

Participants felt very comfortable with the instructor. This undoubtedly facilitated their learning and was important for their enjoyment.

*“And she wasn’t rattling off the information and she’s an incredibly patient person and no, I look forward to it [*…*]I don’t* ***realise*** *I’ve learnt a lot and you know, until she’ll say something, and I think, oh yes, I know what you mean” (P001, Age 76)*.

In this unfamiliar learning environment, and with unfamiliar technology, participants often felt unable to articulate their digital needs or struggles. In an online environment it was more difficult for them to show the instructor the issue, and consequently, they could feel ‘flustered’ and uncomfortable. Participants described how the instructor’s patience helped them feel more comfortable when learning in this environment, and with unfamiliar equipment.

“She was so patient and understanding and the bits that I was not understanding, and you know, fumbling about and not being able to change pages and everything. She understood what my problem was, and she was able to help me” (P015, Age 88).

#### The digital vs. in-person environment

It was clear participants presented individual differences and needs in terms of the learning environment. Whilst participants appreciated the changes to the running of the WW.it programme from in-person, group sessions, to online, one-to-one sessions, through the COVID-19 pandemic; there was an awareness of limitations and, for some, a preference for face-to-face training moving forward.

*“Well I think the training really was difficult. I think I would really like somebody who comes to my house” (P020, Age 79)*.

For some, online learning was daunting as they were unfamiliar with the online environment.

*“In the way that we’ve had to deliver the training. I mean I’m doing 99% of the training online one to one. [*…*]I mean a lot of these ladies had never, never been online before in their lives, never mind suddenly doing a training session over a video call, online” (Instructor)*.

Once more, the instructor’s skills were fundamental to this method of training, which was “*as good as it could be under the circumstances”* (P016, Age 79).

*“And how she tackled it at a distance [*…*]I couldn’t necessarily explain to her all the time what was showing on my screen. So, I was having to hold up, you know, my computer so she could see what I could see, and she would do that [*…*] for an hour at a time, an hour and a quarter [*…*]. It must have been absolutely draining for her mentally, but I mean, she was so patient and so good sorting things out for me” (P016, Age 79*).

Participants made suggestions of how the online, remote training sessions could be further improved. For example, an aide memoir, or a programme handbook, to accompany sessions and facilitate remembering content during and in-between sessions.

*“Just something simple, points, you know, if you want an attachment for example, you know, this is what you do” (P010, Age 76)*.

This was suggested as a way of remembering the content of the session and as something to look at in-between sessions.

*“If we’d also had access, perhaps even on the website in the members area, like an aid memoire where you can go on there because after, I think it as an hour, I had with [anonymized] [*…*] it was quite intense, and we covered a huge amount, but then as you are starting to do things as the weeks go on, you think, now what did she say? [*…*] which keys do I press?” (P032, Age 71)*.

Some regretted not having taking notes during their training session to refer to when practising at home alone.

*“The only thing I regret about it, I didn’t think to take a pen and a paper with me to write down, there and then so I could sit and look at it and say now remember this bit, what happened there and why did you do it and now do it” (P015, Age 88)*.

Participants also recommended shorter, more frequent sessions, as opposed to one 2 h session, feeling this would aid learning and allow them to practice skills in-between sessions.

*“Well, I would suggest that you did it in sound bites. Instead of a whole 2 h all at once [*…*] Teach somebody one thing maybe over 10 min and then tell them to practice” (P021, Age 78)*.

The repetition and opportunity to practice may have supported skills development and retention.

*“It’s something I haven’t retained because I haven’t used it and you need repetition to do that” (P026, Age 66)*.

This would also have given participants the chance to ask questions when the next session resumed.

*“It was fairly intense, and you had to keep up with it [*…*] it’s not always easy to do that in a very limited amount of time with something new. You often need to pause, think about it. Make sure you’ve understood it and then ask any questions if you’ve got any, but obviously that wasn’t possible” (P032, Age 71)*.

Group, face-to-face sessions were considered potential opportunities of sharing with peers, which was not possible when online and in a one-to-one setting.

*“Doing it face to face or even if it was possible to join a few of us together in one area. And you get [*…*] a connection there, you know, before you do anything else and then you learn together. And then you keep sharing together and you know, in touch together and then you can ask questions a lot more and yes, you know, you are time limited doing that hour. I mean if it had been more than one session and if it had been face to face, it would have, yes, obviously it would have been probably even more useful” (P032, Age 71)*.

However, some participants did weigh up both pros and cons of group learning, acknowledging the drawbacks to doing this as a group, such as through reduced session personalization.

*“I do appreciate individually you would get more attention from the trainer”* (P026, Age 66).

“…*the one to one for me was far more beneficial” (P016, Age 79*).

Secondly, the geographical dispersion of members of the WWA was recognized as a barrier and, as a national programme, would make it difficult to get individuals together in one place.

*“COVID has really changed the entire way that we’ve had to look at the project [*…*] ideally the training was going to be used as a social thing as well and I was going to try and get people from as close as possible together. Which was actually proving to be quite difficult anyway because actually the members that have taken part in the project are very, very far flung and spread anyway” (Instructor)*.

#### Summary

The first theme highlights the importance of personalization in delivering the WW.it programme, in terms of understanding motivations for participation, as well as previous experience and use of technology. Through this, there was a deeper understanding of the barriers to digital use and this understanding assisted in developing the programme to suit the participant’s individual needs. Not only did this help empower the learner and improve accessibility, but this also influenced the perception of the programme. The rapport between the participants and instructor was vital in facilitating learning and how receptive the participant was to learning digital skills, particularly to those who were initially anxious due to limited previous experience.

Participants suggested that future training programmes should occur in a group, face-to-face setting to enable peer-to-peer discussions, however, this could impact the personalization of the programme and could be difficult to achieve due to geographical dispersion. Future online, remote sessions could be further improved by utilizing aide memoirs to facilitate practicing digital skills outside and following completion of the digital skill programme. Further suggestions included shorter, more frequent sessions.

### Encouraging further learning

#### The learning journey

For many participants, the WW.it training was the first digital skills programme they had attended to support their use of digital technology, and it was the first programme all participants had attended which was focused on the iPad. For some, there were barriers that remained which negatively influenced their ongoing use of the iPad.

*“I have to be honest with you, I find it very difficult” (P006, Age 90)*.

The WW.it programme was flexible in that the participants who required further training were able to request this, however, this training was not intended to be continued long-term. Multiple participants discussed the need to contact the instructor with additional queries.

*“[The instructor] has said to me she will be in place, so to speak, until the end of November. So I will send her an email with the question and get an answer to that” (P016, Age 79*).

*“It is a learning curve, it’s quite a steep learning curve, but I haven’t [*…*] the thing that I’ve got to get sorted with her and I will ring her about it is the [*…*] is this other thing, is this email [problem]” (P020, Age 79)*.

For most, the sustained learning needs were through their fresh understanding of the iPad and its potential. Participants picked up basic skills, and some personalized to their needs in the WW.it programme, but they discussed areas in which they required continued learning – either formally, informally through friends and family, or through self-led practice and discovery.

*“I did go to the library every day, you get 2 h free at the library. So I could always stay in touch there” (P021, Age 78)*.

*“I have been able to take it down to the local library and get on to the internet connection thing down there and another one in the coffee shop in town” (P023, Age 71)*.

*“So, there are just things that I keep discovering that are out there which perhaps I didn’t really use or know about really” (P032, Age 71)*.

The programme was a launchpad for participants’ learning and it is evident that further learning was needed as time moved on. This must be taken into consideration when considering similar programmes in the future.

Whilst participants were engaged in the WW.it programme for a specific period of time, they discussed their intentions of sustained learning through informal networks (i.e., family and friends) or formal digital skills training programmes. However, methods of sustained learning were not always positive or well-received. It was clear that family support was important for technology use, and in some cases, family members gave them devices as a gift.

*“Just a few months ago my son gave me, is it called an iPad? And he showed me how I could read the Daily Mail” (P015, Age 88)*.

*“I’ve got a few nieces. My other niece has*… *is quite savvy and she said, bring your tablet round and we will have, every Tuesday, because I go to her house every Tuesday for dinner” (P021, Age 78)*.

Relying on family members for ongoing support was not always straightforward, however, and some participants were concerned about seeking help and being seen as a burden. Some described the guilt they felt from asking their family for help, partly through time constraints.

*“But you see, if you had the grandchildren and children, they haven’t necessarily got the time to teach you, because people are so busy working” (P001, Age 76)*.

*“I don’t progress very much because I don’t want to keep going pestering my son saying, how do you find this, how do you find that?” (P015, Age 88)*.

Some also described feeling ‘stupid’ or acknowledged their family’s frustration when trying to support them with technology.

*“[My family have] given up with me. They think I’m such an idiot that trying to explain technology to me is a waste of time. So they’ve given up” (P026, Age 66)*.

However, for some, the support provided by family members was perceived to be inadequate. It was suggested that some children did not have the patience to demonstrate how to use certain functions or make assumptions about their skills.

*“But then they fit us up with the technology, but they assume that we will know how to use it” (P010, Age 76)*.

*“She just tells me, oh mum, I can do it quicker and then, so she does it for me” (P018, Age 80)*.

Not all participants had family to support their continued learning, and this is important to consider within future digital learning programmes, especially when considering peer support that may be offered from group sessions.

*“See I don’t have children. I don’t have nieces and nephews handy. There’s no sort of 12 years-old I can go to” (P016, Age 79*).

One participant, reflected on family dynamics for those who were aging without children, and commented that peers suggest asking their children or grandchildren. It was felt that there was an assumption that they had a family, and that family would be able to help.

*“So yes, all of us don’t have family. So, we don’t actually*… *They say go and ask your grandchild. We don’t have them to ask” (P001, Age 76)*.

Some participants sought out peer support from friends and neighbors.

*“I have to try and get other people to help me [*…*] I have a new next-door neighbour and she’ll sort of come in” (P020, Age 79)*.

*“He is the son of someone who used to live in the same street. He is very, very good and he is retired so you know he is free in the daytime. But of course, at the moment we can’t do anything because we are not allowed[*…*]” (P016, Age 79)*.

Of course, this support was not always available, as discussed by P016, above, in the context of COVID-19 social restrictions.

The WW.it programme supported ongoing personal development. Whilst this formal sustained practice was sought through the WW.it programme, and was intended to supplement this, barriers related to both COVID-19 and geography meant that this was not possible.

*“I mean I had already started making links with charities and what not [*…*] some charities were like, oh we’d love to help you, but [*…*] we don’t have anybody in our area [*…*] There is a charity that does have digital champions and they cover the UK and I thought, great, I am glad I found you, but again they’re still trying to find volunteers to cover lots of areas. So still they have quite a few areas where our War Widows are, that they don’t have anybody” (Instructor)*.

Importantly, participants were not always on the receiving end of this training and demonstrated a genuine desire to empower their peers. P007 reflected on how many of her peers have not been given adequate training and discussed her plans to share her knowledge and skills after the WW.it training. This was a positive, and unanticipated ripple effect from the project.

*“I have people already who are queuing up now who want me to train them on iPads they have had for ages in a drawer, because their family members don’t get round to showing them how to use the device to its full potential” (P007, Age 88)*.

#### Wider barriers to online participation

Despite the want, and need, for further digital skills training, there were barriers to accessing or engaging with digital technology. These were both community-specific factors, including COVID-19 restrictions and lack of access to digital technology outside of the home, and personal factors including self-perceived aging stereotypes and health status which hindered individuals’ own perceived autonomy of using digital devices and increased feelings of vulnerability.

COVID-19 had an extraordinary impact on digital technology use within their own daily living. Some were left feeling “*vulnerable*” *(P001, Age 76)* through digital exclusion.

*“You had to be online, and I realized that I wasn’t” (P001, Age 76)*.

Restrictions associated with the pandemic also halted the support individuals received from community organizations.

*“until we had the pandemic, anything I was stuck on I used to pop down to our local library which has computers and get their advice, but of course that is missing now” (P010, Age 76)*.

The restrictions caused by the pandemic meant that individuals had no way to access or engage with digital technology outside of their own home until they were enrolled onto the WW.it programme. Not only did COVID-19 negatively influence digital technology use in the community, but also restricted support received from friends and family who were unable to visit.

Participants considered their own aging as a barrier which hindered digital access and use. Some used self-deprecating terms such as being a “*dinosaur*” *(P026, Age 66)* when discussing their lack of digital awareness or skills. These increased perceptions of intergenerational ‘othering’ where younger generations were more digitally aware, engaged and capable than themselves.

*“And they are getting left behind and if you get left behind, youngsters think you’re a thicko, you know, you’ve got not brains” (P021, Age 78)*.

Participants discussed how people of their generation are not particularly engaged with technology, unless through the encouragement from younger family members.

*“I don’t think people of our generation are really but I mean we’ve got them. Really thanks to our children’s efforts sort of saying, you’ve got to be able to do this and you’ve got to be able to do that” (P010, Age 76)*.

Consideration was also given to the biological impact of aging and how this can impact the learning process.

*“And as you get old, because I am 87. It does take you longer to absorb things” (P022, Age 88)*.

Participants’ health status, including dexterity problems and eyesight issues, also influenced their use of technology.

*“But you see my hands are getting slower and with arthritis you hit the wrong buttons” (P022, Age 88)*.

*“I found Windows difficult because of the [*…*] the glare from the screen and my contact lens woman gave me a shield, but it made it so dark I couldn’t see it. I couldn’t see the screen” (P001, Age 76)*.

#### Summary

*Encouraging further learning* explored the value of long-term training and learning following the programme completion. This continuation of the learning journey was achieved formally through other training programmes, informally via friends and family, or was self-led. Whilst informal peer networks were valuable in receiving support with queries and encouraging further learning, there were barriers to seeking this support, such as feeling like a burden. Further barriers to continued learning included geographical restraints and the continuation of the COVID-19 pandemic which was occurring at time of data collection.

The subtheme *wider barriers to online participation* noted the influence of community-specific factors and self-perceived aging stereotypes and health status. The COVID-19 pandemic and resultant national lockdowns resulted in feelings of vulnerability and prevented access to previously available community support networks. Self-perceived stereotypes regarding age and abilities, along with health concerns, also impeded access to further learning.

## Discussion

This study aimed to explore the perceived facilitators and barriers of the online digital skills programme, WW.it, and to reflect remote, online, one-to-one training as a possible alternative to traditional face-to-face models.

Unintentionally, this study has become one of the first studies to evaluate online digital skills delivery for older adults (females) in the UK during the COVID-19 pandemic and national lockdowns. This model for digital skills training was unique, utilizing remote, one-to-one digital skills delivery to individuals who had very little to no previous experience of using technology. Through this unique implementation and delivery, there were clear identified elements of training and lessons learned for future implementation. Furthermore, it is recognized that this training programme sits in the wider context and relies on other elements to sustain reach and impact.

Personalized content through familiarization between the instructor and learner, and one-to-one learning, was a major benefit of the WW.it programme and enabled participant empowerment. Learners described their anxieties in beginning training however, the relationship they had with the instructor eased concerns. Building a positive relationship with older adults enhances learning (Wlodkowski, 1999). Evidence demonstrates the ineffectiveness of non-personalized ICT courses in supporting older adults to get online (Age UK, 2018). The personalized approach taken within the WW.it programme enabled the instructor to recognize individualized content and individual learning needs. These are both key components of the Critical Geragogy learning theory (Findsen and Formosa, 2012), and are reflected in the digital skills reflective tool for older adults (Gates and Wilson-Menzfeld, 2022). Acknowledging these issues can reduce perceived barriers or accessibility needs (Seo et al., 2019).

It is beneficial when learning is relatable and mimics real world scenarios (Peterson, 1983). Personalized learning can support this learning style, enhancing the value an individual sees from digital technology (Brown and Strommen, 2018; Seo et al., 2019; LoBuono et al., 2020). The recognition of the tangible outcomes from using the internet is one component of digital exclusion (Van Deursen and Helsper, 2015). Many participants did not recognize the potential benefits of the iPad or the internet as they had little to no prior experience of use, as is recognized in wider literature (Tsai et al., 2015), however, through personalization of this digital skills programme, they were supported to use this device for their own interests and consequently learned how this technology could benefit themselves, and their own daily living. Whilst instructors, and their relationship with each learner, differ between programmes and cannot necessarily be replicated, the importance of developing a relationship with learners, and the consistency of an instructor on a digital skills programme, is recommended from this study. However, it is important to consider the practicalities and sustainability of this method on a larger scale. This relationship building and understanding of an individual’s needs took time which may not be possible depending upon funding, programme length, and group-based support.

Despite some of the advantages to utilizing online, remote learning, participants sometimes found it difficult to learn digital skills remotely and consequently still preferred face-to-face, group learning. The social advantages of group learning are notable. Group settings encourage peer learning and support, enhance social inclusion (Wlodkowski, 1999), and increase confidence (Zaidman and Tinker, 2016). Vygotsky (1978) suggested that whilst individual learning could increase knowledge and skills, collaboration with more informed peers could further enhance this and expand the zone of proximal development, the distance between current developmental stage and maximum potential development. This could indicate that knowledge of digital skills could be further enhanced through interaction and learning with peers.

When suggesting improvements for the WW.it programme and the online learning environment, participants made various suggestions for additional learning materials, such as an aide-memoire, along with shorter, more frequent sessions. Flexibility, pace, repetition, and reflection are some of the good-practice principles suggested to engage older adults in technology use (Age UK, 2018; Centre for Ageing Better, 2018) and, once more, reflect key components of learning theory (Formosa, 2002, 2012). The use of additional materials, or adapted session layouts, may have supported continued learning after the training had ended. Remote, online digital skills learning can be advantageous for various reasons, including inclusivity, accessibility, and cost, however, it is not preferred by all learners. It is critical to consider the needs of learners and examine the benefits or drawbacks of this approach before implementation.

Learning does not only occur in one discreet setting. It was evident that individuals were empowered to continue learning informally outside of sessions, through friends and family members. For many participants, the WW.it programme was just the beginning, and many wanted to continue partaking in other formal digital skills training sessions to further improve their skills. There were however, some barriers to digital skills learning, both as part of the WW.it programme and further learning.

All participants in this study were female and over 65 years old, and perceptions of their own aging was a barrier to their digital learning. Aging stereotypes are considered as being a barrier in Critical Geragogy (Findsen and Formosa, 2012) and evidence demonstrates the power of self-considered aging stereotypes and low self-efficacy in learning (Neves et al., 2013; Wilson et al., 2021). These self-perpetuating views can be exacerbated through prior negative experiences of learning new digital skills (Centre for Ageing Better, 2018). Critical Geragogy and the reflective tool for the delivery of digital skills for older adults (Formosa, 2012; Gates and Wilson-Menzfeld, 2022) promotes the importance of challenging negative perceptions of aging, across both learners and instructors. By challenging these assumptions and encouraging self-efficacy, learners are more likely to have the willingness to use digital services.

Additionally, individuals perceived their own health as a barrier to digital skills learning through lack of accessibility, as recognized in the evidence base (Neves et al., 2013; Wilson et al., 2021). In this study, health needs predominantly included eyesight and dexterity issues. Critical Geragogy recognizes the different physical, as well as emotional and social needs of older adults and something that needs to be considered in the learning environment (Formosa, 2018; Hunsaker and Hargittai, 2018).

### Strengths and limitations

Through examining remote, online digital skills training for older adults, a strength of this study is its original contribution to knowledge in the field of digital inclusion. However, there are also limitations to this research. Due to the nature of WWA membership, this sample consisted of all white females, over 65 years old. Whilst this represented the organization in which the WW.it programme was implemented, it limits how the findings can be translated to the wider population. Furthermore, the voluntary nature of study sampling means that the study included individuals who were interested in technology. Whilst, arguably, anyone participating in digital skills training, as part of research or not, is self-selecting, this voluntary sample is not representative of all perspectives on digital learning and technology use.

### Recommendations for practice

The WW.it programme did not utilize the reflective tool for delivering digital skills to older female participants from the offset, and it is recommended for future programmes to do so to optimize the learning environment.

One recommendation from this study is to consider the importance of learning theories, in this context Critical Gerontology, when designing and delivering digital skills programmes. For example, through recognizing the importance of self-held aging stereotypes on learning, and placing emphasis on individual learning styles, through use of the reflective tool for delivering digital skills to older adults. Multiple practical recommendations for digital skills training also arose from this study and are recommended for consideration in future delivery programmes aimed at older adults, for example, shorter sessions spread across several weeks, face-to-face, group classes (where possible), additional materials to accompany training, a focus on accessibility settings, and personalized learning and content. The consistency of instructor and relationship building should be a priority when planning digital skills training programmes.

Finally, signposting information should be provided by organizations for learners to seek further training once programmes are completed, with the recognition of wider barriers of digital use, such as access inside and outside of the home. This could be through local digital champions, national digital organizations, textbooks, or online-only resources.

### Recommendations for future research

Research that considers online digital skills training programmes is still in its early stages and further work is needed to expand the evidence in this area. Further research is also needed to examine the impact and effectiveness of digital learning in a group setting as this appears to be scarce. This study acknowledges the homogeneity of its sample and further research must consider the inclusion of wider cohorts to be more reflective of the wider, older population. Future research in this area should utilize the reflective tool for delivering digital skills to older adults (Gates and Wilson-Menzfeld, 2022) when evaluating programmes to improve digital skills, as this is the first study to do this.

## Conclusion

The WW.it programme aimed to empower older women through digital inclusion, and to support the development of new skills to connect with others online, by providing iPads and/or iPad training.

Due to the COVID-19 pandemic, this programme took an unexpected and unique route to delivering these digital skills through online platforms. This paper has reflected on the remote, online, one-to-one training as an alternative to face-to-face models, and has explored perceived facilitators and barriers of the programme. The participants in this study were new to the digital sphere, and due to the pandemic, had to learn digital skills using an unfamiliar and online format. While this online environment isn’t suitable for all, there were benefits to this mode of delivery, such as a personalized approach that was valued by participants. This study also emphasizes the importance of a developing the instructor-learner relationship.

Participants developed basic skills, personalized to individual needs, and many sought additional learning through formal training, informal assistance by family or friends, or self-led practice. However, some of these avenues were limited due to COVID-19 restrictions, fears of being a burden, or lack of access to familial support. Self-perceived aging stereotypes and health issues could impede perceived autonomy of using digital devices.

This programme exhibited the ethos and principles of Geragogy and Critical Geragogy, and while undoubtedly encountered barriers, it promoted the empowerment of participants to learn relevant digital skills through a programme tailored to suit their needs.

## Data availability statement

The datasets presented in this article are not readily available because participants have not consented for full interview transcripts to be made publicly available. Requests to access the datasets should be directed to GW-M, gemma.wilson-menzfeld@northumbria.ac.uk.

## Ethics statement

The studies involving human participants were reviewed and approved by Northumbria University Health and Life Sciences Ethics Committee (ref: 120.3305). The patients/participants provided their written informed consent to participate in this study.

## Author contributions

GW-M study PI and oversaw the running of the whole project. GW-M, HR, and JG completed participant recruitment. GW-M, JG, MM, and AJ completed qualitative and quantitative data collection and/or analysis for both stages of the project. GW-M led on writing the submitted manuscript with AJ, JG, and MM. HR contributed to the writing sections. All authors contributed to the article and approved the submitted version.

## Footnotes

1 There are several possible explanations for the variation in interview times. For instance, this is likely due to prior limited knowledge of digital technology and what to expect from participating in the WW.it programme (explored in Phase One interviews). Additionally, it is likely that the participants were experiencing high levels of loneliness and social isolation, particularly as most of these interviews took place during the COVID-19 pandemic and, given the age of these participants, they were considered an at risk group and recommended to self-isolate.
