# Corrigendum: Learning digital skills online: empowering older adults through one-to-one, online digital training provision

**DOI:** 10.3389/fpsyg.2023.1339578

**Published:** 2023-12-08

**Authors:** Gemma Wilson-Menzfeld, Jessica Raven Gates, Mary Moreland, Helen Raw, Amy Johnson

**Affiliations:** ^1^Department of Nursing, Midwifery, and Health, Faculty of Health and Life Sciences, Northumbria University, Newcastle upon Tyne, United Kingdom; ^2^War Widows' Association of Great Britain, London, United Kingdom

**Keywords:** digital, critical geragogy, older adult, skill building, digital skill development, facilitators, barriers, training

In the published article, “Braun and Clarke, 2006” was incorrectly referenced in some parts of the text, outlined below.

- The WWA recognized issues of loneliness and social isolation across their membership, along with the desire of members to be connected to other members throughout the UK, Working with (Institution), the WWA designed a digital intervention, the War Widows InTouch (WW.it) programme, to address these needs and to connect older war widow(er)s (over 65 years old) at both a national and local level (Braun and Clarke, 2006).
- Utilizing Critical Geragogy as an underpinning learning theory, WW.it aimed to provide a personalized intervention which encouraged older war widows to take an active role in digital skills training, working collaboratively with the instructor throughout (Braun and Clarke, 2006).
- To accomplish this, members of the WWA were given iPads and/or iPad training (Braun and Clarke, 2006).
- This mixed methods design, underpinned by Pragmatism (Feilzer, 2010; Morgan, 2014), allowed the research team to identify the self-reported impact of the WW.it programme, whilst gathering in-depth information regarding the implementation (see Braun and Clarke, 2006 for full evaluation).

Instead of “Braun and Clarke, 2006,” “Wilson-Menzfeld et al., 2021” should be cited in these instances, linked to the following reference: Wilson-Menzfeld, G., Gates, J., Johnson, J., Moreland, M., and Raw, H. (2021). *Exploring and Evaluating the War Widows InTouch (WW.it) Programme*. Newcastle-upon-Tyne: Northumbria University.

Additionally, in the Introduction, the word “Institution” in the text “Working with (Institution)”, and in the Materials and Methods, Design, in the text “Ethical approval was received from (Institution)'s ethical approval system”, should have been replaced with the name of the institution following peer review. “(Institution)” should be replaced with “Northumbria University”.

The following corrections have been made to the article.

Citation correction has been made to *Introduction, Paragraph 7*. The corrected paragraph is shown below.

The War Widows' Association (WWA) is a registered charity with 1,941 members (as of November 2021). To be a full member, an individual must receive/have received a War Widows' Pension or Armed Forces Compensation Scheme 2005 payments. Any individual interested in the welfare of War Widow(er)s or in supporting the aims of the WWA can become an associate member. The WWA recognized issues of loneliness and social isolation across their membership, along with the desire of members to be connected to other members throughout the UK. Working with Northumbria University, the WWA designed a digital intervention, the War Widows InTouch (WW.it) programme, to address these needs and to connect older war widow(er)s (over 65 years old) at both a national and local level (Wilson-Menzfeld et al., 2021). The WW.it programme also aimed to increase digital access, digital confidence, and digital skills, as well as reducing fear and the impact of aging stereotypes on digital learning. Utilizing Critical Geragogy as an underpinning learning theory, WW.it aimed to provide a personalized intervention which encouraged older war widows to take an active role in digital skills training, working collaboratively with the instructor throughout (Wilson-Menzfeld et al., 2021). To accomplish this, members of the WWA were given iPads and/or iPad training (Wilson-Menzfeld et al., 2021). This project took lessons from “Project Semaphore” which was carried out by the Royal Naval Association and had similar project aims (Royal Naval Association, n.d.). However, due to the COVID-19 pandemic, the implementation and running of the WW.it programme changed significantly. Initially the programme was intended to be completed face-to-face, and in a group setting, but was ran remotely, online, and in a one-to-one setting. This training model allowed individuals to receive digital skills training at a time when the use of technology was being perceived as a fundamental part of everyday life. However, this is a very different model of training than had been previously considered.

A correction has also been made to *Materials and Methods, Design*. The corrected section is shown below.

This study is part of a larger, two-phase project which involved a mixed-method explanatory sequential design (Creswell et al., 2011). Mixed methods designs are typically chosen for evaluation studies to assess the impact of a programme, whilst also providing an in-depth view of the participant experiences to provide a more complete picture (Creswell and Clark, 2017). This mixed methods design, underpinned by Pragmatism (Feilzer, 2010; Morgan, 2014), allowed the research team to identify the self-reported impact of the WW.it programme, whilst gathering in-depth information regarding the implementation (see Wilson-Menzfeld et al., 2021 for full evaluation). This paper will focus on the data collected as part of semi-structured interviews across both Phase One and Phase Two only. Quantitative analysis from this mixed methods study is presented elsewhere (Wilson-Menzfeld et al., 2021). Ethical approval was received from Northumbria University's ethical approval system (ref: 120.3305). This study adhered to the UK Government's COVID-19 rules and Northumbria University's guidance on social distancing and completing face-to-face research.

The authors apologize for these errors and state that they do not change the scientific conclusions of the article in any way. The original article has been updated.

